# Refining gastric cancer staging: examining the interplay between number and anatomical location of metastatic lymph nodes - a retrospective multi-institutional study

**DOI:** 10.1186/s12885-023-11653-0

**Published:** 2023-12-05

**Authors:** Chul-Hyo Jeon, Ki Bum Park, Hayemin Lee, Dong Jin Kim, Ho Seok Seo, Junhyun Lee, Kyung Hwa Jun, Jin Jo Kim, Han Hong Lee

**Affiliations:** 1 Division of Gastrointestinal Surgery, Department of Surgery, Uijeongbu St. Mary’s Hospital, College of Medicine, The Catholic University of Korea, 271, Cheonbo-Ro, Uijeongbu-si, Gyeonggi-do, 11765 Republic of Korea; 2 Division of Gastrointestinal Surgery, Department of Surgery, St. Vincent’s Hospital, College of Medicine, The Catholic University of Korea, 93, Jungbu-daero, Paldal-gu, Suwon-si, Gyeonggi-do, 16247 Republic of Korea; 3 Division of Gastrointestinal Surgery, Department of Surgery, Bucheon St. Mary’s Hospital, College of Medicine, The Catholic University of Korea, 327, Sosa-ro, Wonmi-gu, Bucheon-si, Gyeonggi-do, 14647 Republic of Korea; 4 Division of Gastrointestinal Surgery, Department of Surgery, Eunpyeong St. Mary’s Hospital, College of Medicine, The Catholic University of Korea, 1021, Tongil-ro, Eunpyeong-gu, Seoul, 03312 Republic of Korea; 5 Division of Gastrointestinal Surgery, Department of Surgery, Seoul St. Mary’s Hospital, College of Medicine, The Catholic University of Korea, 222, Banpo-daero, Seocho-gu, Seoul, 06591 Republic of Korea; 6 Division of Gastrointestinal Surgery, Department of Surgery, Incheon St. Mary’s Hospital, College of Medicine, The Catholic University of Korea, 56, Dongsu-ro, Bupyeong-gu, Incheon, 21431 Republic of Korea

**Keywords:** TNM, Staging system, Gastric cancer, Nodal stage, Metastatic lymph nodes stomach neoplasms, Gastrectomy, Lymph node excision

## Abstract

**Background:**

The current gastric cancer staging system relies on the number of metastatic lymph nodes (MLNs) for nodal stage determination. However, incorporating additional information such as topographic status may help address uncertainties. This study evaluated the appropriateness of the current staging system and relative significance of MLNs based on their anatomical location.

**Methods:**

Patients who underwent curative gastrectomy for gastric cancer between 2000 and 2019 at six Catholic Medical Center-affiliated hospitals were included. Lymph node-positive patients were classified into the perigastric (stations 1–6, group P) or extragastric (stations 7–12) groups. The extragastric group was further subdivided into the near-extragastric (stations 7–9, group NE) and far-extragastric (stations 10–12, group FE) groups.

**Results:**

We analyzed the data of 3,591 patients with positive lymph node metastases. No significant survival differences were found between group P and the extragastric group in each N stage. However, in N1 and N2, group FE showed significantly worse survival than the other groups (*p* = 0.013 for N1, *p* < 0.001 for N2), but not in N3. In the subgroup analysis, group FE had a significantly lower overall survival in N2, regardless of the cancer location.

**Conclusions:**

Our large-scale multi-institutional big data analysis confirmed the superiority of the current numerical nodal staging system for gastric cancer. Nonetheless, in N1 and N2 in which there is an upper limit on metastatic nodes, attention should be paid to the potential significance of topographic information for specific nodal stations.

**Supplementary Information:**

The online version contains supplementary material available at 10.1186/s12885-023-11653-0.

## Introduction

Gastric cancer (GC) remains a significant public health problem worldwide as it is ranked fifth for incidence and fourth for mortality worldwide. GC accounted for over one million new cases and 769,000 deaths in 2020 [1]. In Korea, there is a marked decline in the incidence of GC and an increasing prevalence in early GC [2]. Nevertheless, curative gastrectomy and postoperative adjuvant chemotherapy are considered the mainstay of treatment for GC [3].

The Tumor–Node–Metastasis (TNM) staging system is used to evaluate the prognosis of patients with GC and to determine optimal treatment options based on disease severity [4]. Particularly, the intelligibility and conciseness of the numeric N stage have been improved to provide a more convenient means of predicting prognosis and making objective comparisons with previous anatomical-based classifications [5–8]. Although previous studies have reported the prognostic superiority of the numeric N system over the topographical system in GC [9], it has some limitations: uncertain cut-offs for the optimal N stage, insufficient anatomical information regarding the extent of lymph node metastasis (LNM), and the surgical extent of lymphadenectomy. Additionally, the current system proves disadvantageous as it does not reflect the complexity and multidirectional structure of the perigastric lymphatic drainage pathways [10].

Therefore, using a large-scale dataset, we investigated the appropriateness of the current nodal staging system based on the number of metastatic lymph nodes (MLNs). Moreover, by considering the relative significance of MLNs according to location, we aimed to identify ways to address the limitations of the current staging system and enhance the prognostic performance through a comprehensive big data analysis.

## Materials and methods

### Patient population and data collection

**Fig. 1:**
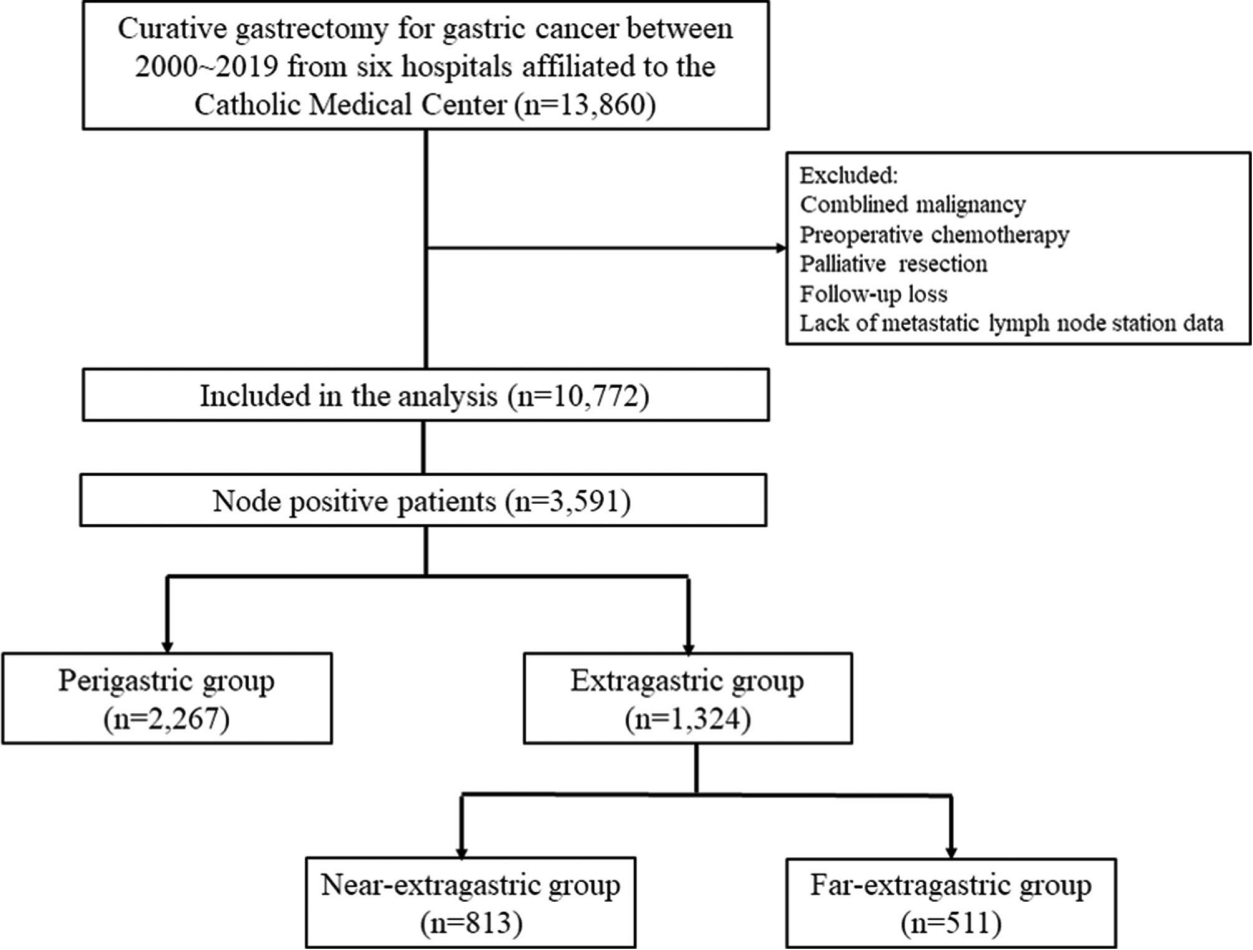
Flowchart depicting the patient enrollment process of the study cohort

In total, 13,860 patients who underwent curative gastrectomy with lymphadenectomy for GC between January 2000 and December 2019 at six hospitals affiliated to the Catholic Medical Center in Korea were recruited: Seoul St. Mary’s Hospital (*n* = 6,591), St. Vincent’s Hospital (*n* = 1,524), Incheon St. Mary’s Hospital (*n* = 1,437), Bucheon St. Mary’s Hospital (*n* = 956), Yeouido St. Mary’s Hospital (*n* = 764), and Uijeongbu St. Mary’s Hospital (*n* = 634). The inclusion criteria were as follows: primary GC, no other malignancy, no preoperative chemotherapy, no distant metastasis, R0 resection (no residual macroscopic or microscopic tumor), regular outpatient follow-up without disease, and complete data. Patients with missing operative and/or follow-up data were excluded. Finally, 10,772 patients were enrolled. The enrollment process is shown in Fig. 1.

### Surgical details and follow-up

Surgeons specializing in GC performed all surgeries based on the Korean and Japanese guidelines for Gastric Cancer [11, 12]. Patient demographic data were also collected. Preoperative clinical characteristics and postoperative complications were classified using the Eastern Cooperative Oncology Group [13] and Clavien–Dindo criteria [14]. Histological staging was performed according to the 8th American Joint Committee on Cancer TNM guidelines [15]. Histological types were categorized as differentiated or undifferentiated. Poorly differentiated tubular and signet ring cells and mucinous adenocarcinomas were considered undifferentiated. Regular follow-ups were scheduled at 3- and 6-month intervals for patients with advanced and early GC, respectively, for the first 3 years, and every 12 months thereafter. At each follow-up, tumor marker levels were measured, and abdominal computed tomography and endoscopy were performed. The observation period was defined as the time from the date of surgery to the time of death or loss to follow-up, whichever occurred first. Overall survival (OS) was calculated from the date of primary gastrectomy to the date of death from any cause or at the time of the last follow-up.

**Fig. 2:**
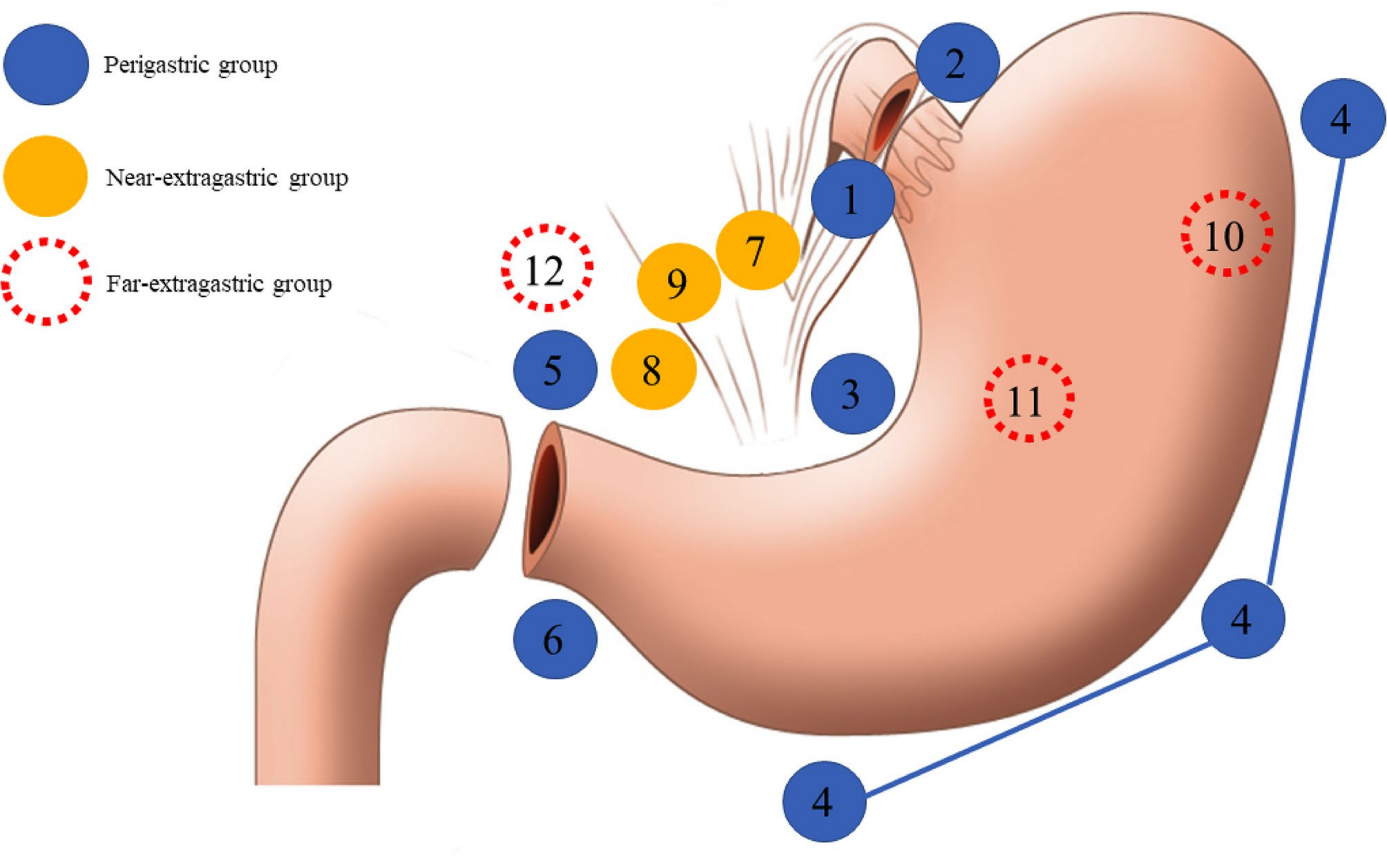
Schematic diagram of the metastatic lymph nodes. The perigastric group had lymph node metastasis (LNMs) in the perigastric region (stations No. 1, 2, 3, 4, 5, and 6). The near-extragastric group included LNMs near the celiac axis (stations No. 7, 8a, and 9). The far-extragastric group comprised stations No. 10, 11, and 12

### Histopathological analysis and categorization of metastatic lymph nodes according to anatomical regions

The specimens were removed via en bloc dissection. Each lymph node (LN) was accurately mapped and harvested either intraoperatively or immediately after surgery via back-table dissection. Histological evaluation was performed by gastrointestinal neoplasia specialists; they providing detailed pathological results according to LN stations.

The pathological findings were used to classify the MLNs. The MLNs were first categorized into the perigastric (group P) and extragastric groups. The extragastric groups were further divided into the near-extragastric (NE) and far-extragastric (FE) groups, altering the previous classification of LN tiers [16, 17] (Fig. 2). Group P had LNMs in the perigastric region (station No. 1, 2, 3, 4, 5, and 6). Group NE included LNMs near the celiac axis (No. 7, 8a, and 9). Finally, group FE comprised stations 10, 11, and 12.

### Statistical analysis

Kaplan–Meier analysis was performed to evaluate OS. Survival data are presented as mean patient survival, as it was not always possible to calculate the median survival. Continuous values are presented as means with standard deviations; they were compared using the Student’s t-test. Categorical variables were compared using the chi-square or Fisher’s exact test (as appropriate). Statistical significance was set at *p* < 0.05. 3. All analyses were performed using SPSS ver. 24.0 for Windows (IBM Corp., Armonk, NY, USA).

## Results

### Patient demographic and clinicopathological characteristics

The patients’ clinicopathological characteristics are shown in Table 1. The total cohort consisted of 7,181 (66.7%) male individuals, with a mean age of 61.16 ± 11.95 years, and a mean body mass index (BMI) of 23.68 ± 3.31 kg/m^2^. Of the 10,772 patients, 2,449 (22.7%) underwent total gastrectomy, 6,880 (63.9%) underwent D2 or higher lymphadenectomy, and 3,591 (33.3%) had with node-positive disease. OS was compared according to pathological stage and revealed a decline in survival with increasing disease severity (Supplementary Fig. 1).

**Table 1:** Patient characteristics according to lymph node metastasis

Variables, *n*(%)	Node (+) (*N* = 3591, 100%)	Total (*N* = 10,772, 100%)	*P*-value
**Age (years)**			< 0.001
< 65	1940 (54.0%)	6268 (58.2%)	
≥ 65	1651 (46.0%)	4504 (41.8%)	
**Sex**			0.371
Male	2423 (67.5%)	7181 (66.7%)	
Female	1168 (32.5%)	3591 (33.3%)	
**ECOG**			< 0.001
0–1	3396 (94.6%)	10,359 (96.2%)	
≥ 2	166 (4.6%)	347 (3.2%)	
N/A	29 (0.8%)	66 (0.6%)	
**Preoperative BMI (kg/m**^**2**^)			< 0.001
< 23	1303 (47.5%)	3611 (42.8%)	
≥ 23	1439 (52.5%)	4834 (57.2%)	
**Approach**			< 0.001
MIS	1222 (34.0%)	5556 (51.6%)	
Open	2254 (62.8%)	4946 (45.9%)	
N/A	115 (3.2%)	270 (2.5%)	
**Resection**			< 0.001
STG	2427 (67.6%)	8261 (76.7%)	
TG	1134 (31.6%)	2449 (22.7%)	
Others	30 (0.8%)	62 (0.6%)	
**Lymphadenectomy**			< 0.001
D1+ ↓	806 (22.4%)	3828 (35.5%)	
D2 ↑	2727 (75.9%)	6780 (62.9%)	
N/A	58 (1.6%)	164 (1.5%)	
**Reconstruction**			< 0.001
B-I	381 (10.6%)	1704 (15.8%)	
B-II	1899 (52.9%)	5758 (53.5%)	
RY	1093 (30.4%)	2752 (25.5%)	
Others	218 (6.1%)	558 5.2%)	
**pT stage**			< 0.001
T1	735 (20.5%)	6448 (59.9%)	
T2	487 (13.6%)	1094 (10.2%)	
T3	1022 (28.5%)	1596 (14.8%)	
T4	1347 (37.5%)	1634 (15.2%)	
**pN stage**			< 0.001
N0	0 (0.0%)	7181 (66.7%)	
N1	1277 (35.6%)	1277 (11.9%)	
N2	972 (27.1%)	972 (9.0%)	
N3a	778 (21.7%)	778 (7.2%)	
N3b	564 (15.7%)	564 (5.2%)	
**pTMN stage**			< 0.001
I	684 (19.0%)	6776 (62.9%)	
II	887 (24.7%)	1796 (16.7%)	
III	2020 (56.3%)	2200 (20.4%)	

*Abbreviations*: *ECOG* Eastern Cooperative Oncology Group performance status, *N/A* not applicable, *BMI* body mass index, *MIS* minimal invasive surgery, *STG* subtotal gastrectomy, *TG* total gastrectomy

**Fig. 3:**
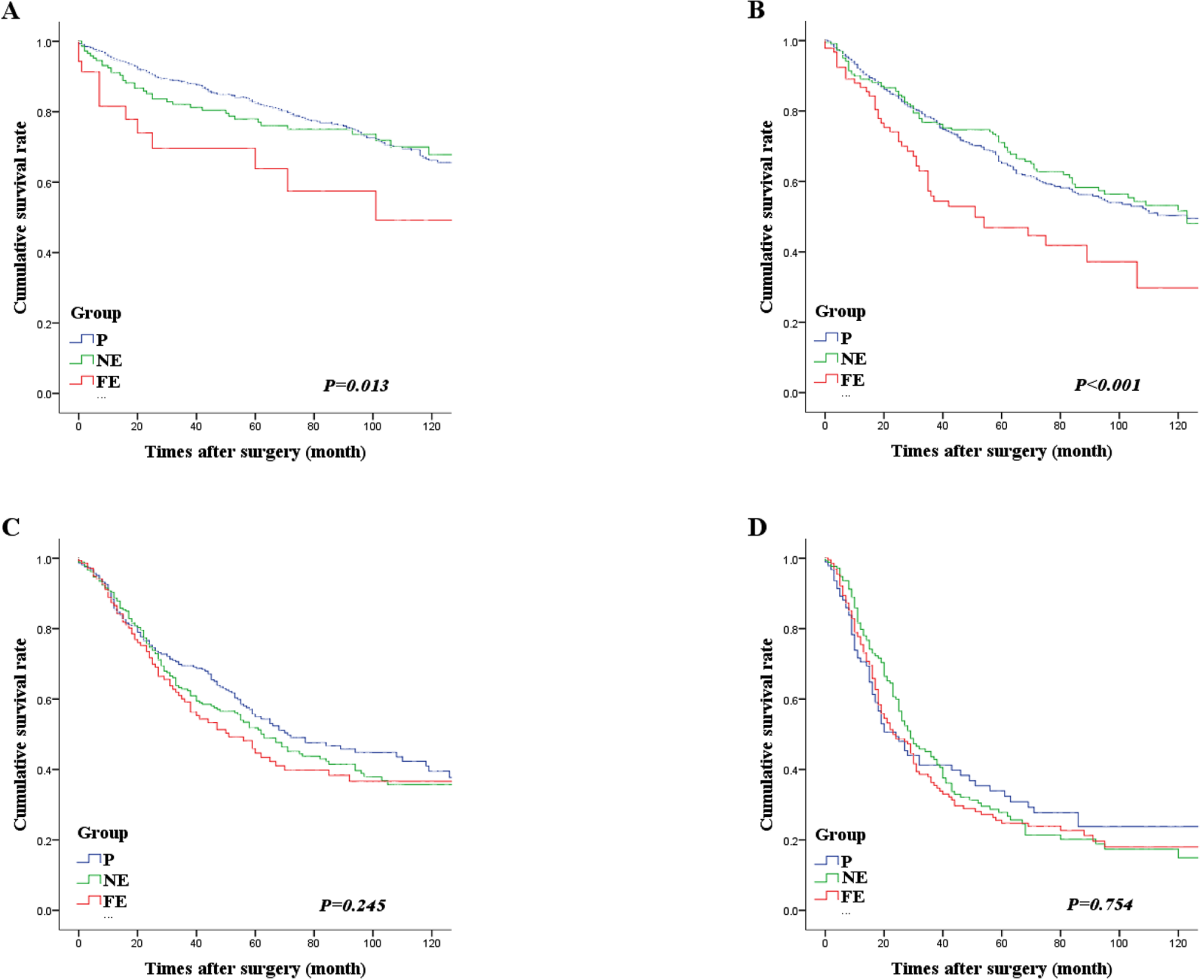
Analysis of the overall survival of the three lymph node metastasis groups according to the N stage. **A**: N1 stage, **B**: N2 stage, **C**: N3a stage, **D**: N3b stage. Patients in the far-extragastric group (group FE) had significantly lower survival rates in N1 and N2 stages

Compared with the total patient group, node-positive patients had a higher proportion of individuals aged 65 years or older, a greater prevalence of patients with a poor performance status, and a lower average BMI. Additionally, these patients underwent more invasive surgeries and radical lymphadenectomies (beyond D2), and had a higher proportion of total gastrectomy cases than in the total cohort. Notably, the node-positive subgroup exhibited a significantly higher disease severity. The sex distribution was comparable between the groups (Table 1).

**Table 2-1:** Multivariate analysis of factors contributing to OS of N1 case

Variables	Univariate			Multivariate		
	HR	95% CI	*p*-value	HR	95% CI	*p*-value
**Older age** (vs. < 65)	2.067	1.632–2.618	< 0.001*	1.545	1.147–2.082	0.004*
**Female sex** (vs. male)	0.554	0.424–0.726	< 0.001*	0.586	0.414–0.827	0.002*
**ECOG 2–4** (vs. 0–1)	6.932	1.624–29.586	< 0.001*	2.372	1.428–3.940	0.001*
**Higher BMI** (vs. <23)	0.653	0.492–0.865	0.003*	0.734	0.551–0.979	0.035*
**Approach** (vs. open)	0.739	0.584–0.935	0.012*	794.021		0.833
**TG (**vs. STG)	0.430	0.158–1.173	0.099			
**Lymphadenectomy (**vs. D1+ ↓)	0.303	0.160–0.573	< 0.001*	0.445	0.229–0.864	0.017*
**T stage**						
T1	Ref			Ref		
T2	1.845	1.229–2.772	0.003*	0.651	0.232–1.825	0.414
T3	3.087	2.216-4.300	< 0.001*	0.954	0.349–2.607	0.927
T4	4.417	3.135–6.222	< 0.001*	0.644	0.277–1.499	0.308
**p TNM stage**						
I	Ref			Ref		
II	2.917	2.116–4.021	< 0.001*	3.435	1.246–9.474	0.017*
III	5.488	3.890–7.742	< 0.001*	8.872	3.723–21.144	< 0.001*
**Group**						
Group P	Ref			Ref		
Group NE	1.144	0.821–1.594	0.428	1.070	0.692–1.653	0.762
Group FE	2.297	1.285–4.106	0.005*	2.457	1.278–4.725	0.007*

*Abbreviations*: *OS* overall survival, *HR* hazards ratio, *CI* confidence interval, *ECOG* Eastern Cooperative Oncology Group performance status, *BMI* body mass index, *TG* total gastrectomy, *STG* subtotal gastrectomy

**Table 2-2:** Multivariate analysis of factors contributing to OS of N2 case

Variables	Univariate			Multivariate		
	HR	95% CI	*p*-value	HR	95% CI	*p*-value
**Older age** (vs. < 65)	1.991	1.615–2.455	< 0.001*	1.676	1.291–2.176	< 0.001*
**Female sex** (vs. male)	0.068	0.640–1.016	0.806			
**ECOG 2–4** (vs. 0–1)	4.703	1.612–13.721	0.005*	0.513	0.308–0.853	0.010*
**Higher BMI** (vs. <23)	0.776	0.605–0.994	0.045*	0.942	0.731–1.215	0.648
**Approach** (vs. open)	1.005	0.586–1.721	0.987			
**TG (**vs. STG)	0.660	0.244–1.790	0.415			
**Lymphadenectomy (**vs. D1+ ↓)	0.379	0.187–0.766	0.007*	1.029	0.741–1.431	0.863
**T stage**						
T1	Ref			Ref		
T2	0.970	0.625–1.508	0.894	0.907	0.296–2.778	0.864
T3	1.571	1.110–2.224	0.011*	0.978	0.306–3.125	0.970
T4	3.067	2.196–4.283	< 0.001*	1.757	1.307–2.364	< 0.001*
**p TNM stage**						
II	Ref			Ref		
III	2.282	1.387–71.778	0.002*	7.154	2.947–18.399	0.002*
**Group**						
Group P	Ref			Ref		
Group NE	0.989	0.773–1.265	0.930	1.399	0.981–1.996	0.063
Group FE	1.775	1.288–2.445	< 0.001*	1.724	1.138–2.611	0.010*

*Abbreviations*: *OS* overall survival, *HR* hazards ratio, *CI* confidence interval, *ECOG* Eastern Cooperative Oncology Group performance status, *BMI* body mass index, *TG* total gastrectomy, *STG* subtotal gastrectomy

**Table 2-3:** Multivariate analysis of factors contributing to OS of N3a case

Variables	Univariate			Multivariate		
	HR	95% CI	*p*-value	HR	95% CI	*p*-value
**Older age** (vs. < 65)	1.538	1.247–1.895	< 0.001*	1.435	1.158–1.778	0.001*
**Female sex** (vs. male)	0.767	0.605–0.972	0.028*	1.190	0.935–1.515	0.158
**ECOG 2–4** (vs. 0–1)	1.202	0.406–3.560	0.740			
**Higher BMI** (vs. <23)	0.828	0.642–1.069	0.147			
**Approach** (vs. open)	1.292	0.802–2.083	0.292			
**TG (**vs. STG)	0.700	0.327–1.497	0.358			
**Lymphadenectomy (**vs. D1+ ↓)	0.883	0.469–1.660	0.699			
**T stage**						
T1	Ref					
T2	1.368	0.787–2.380	0.267			
T3	1.071	0.661–1.734	0.870			
T4	1.908	1.204–3.022	0.006*			
**p TNM stage**						
II	Ref					
III	1.952	1.179–22.146	0.001*	29.442	6.623-264.308	< 0.001*
**Group**						
Group P	Ref			Ref		
Group NE	1.130	0.896–1.426	0.301	1.043	0.824–1.323	0.720
Group FE	1.260	0.950–1.670	0.109	1.024	0.767–1.366	0.873

*Abbreviations*: *OS* overall survival, *HR* hazards ratio, *CI* confidence interval, *ECOG* Eastern Cooperative Oncology Group performance status, *BMI* body mass index, *TG* total gastrectomy, *STG* subtotal gastrectomy

**Table 2-4:** Multivariate analysis of factors contributing to OS of N3b case

Variables	Univariate			Multivariate		
	HR	95% CI	*p*-value	HR	95% CI	*p*-value
**Older age** (vs. < 65)	1.410	1.113–1.786	0.004*	1.146	0.892–1.471	0.286
**Female sex** (vs. male)	1.156	0.901–1.483	0.256			
**ECOG 2–4** (vs. 0–1)	1.205	0.278–5.220	0.804			
**Higher BMI** (vs. <23)	0.911	0.687–1.208	0.518			
**Approach** (vs. open)	0.804	0.578–1.117	0.194			
**TG (**vs. STG)	1.285	1.019–1.622	0.034*	0.778	0.617–0.982	0.034*
**Lymphadenectomy (**vs. D1+ ↓)	1.041	0.462–2.342	0.923			
**T stage**						
T1	Ref					
T2	1.012	0.379–2.699	0.982			
T3	0.953	0.386–2.354	0.917			
T4	1.176	0.483–2.862	0.721			
**p TNM stage**						
III	Ref					
**Group**						
Group P	Ref			Ref		
Group NE	0.982	0.709–1.362	0.914	1.135	0.830–1.555	0.428
Group FE	1.168	0.854–1.598	0.330	1.153	0.884–1.506	0.293

*Abbreviations*: *OS* overall survival, *HR* hazards ratio, *CI* confidence interval, *ECOG* Eastern Cooperative Oncology Group performance status, *BMI* body mass index, *TG* total gastrectomy, *STG* subtotal gastrectomy

### Comparison of survival according to anatomic location of lymph node metastases

#### Comparison of survival between the perigastric and extragastric lymph node metastasis groups across nodal stages

OS was compared between group P and the extragastric groups according to the N stage; there was no significant difference in 5-year survival rates (5YOS) at different nodal stages. In N1, the 5YOS in group P and extragastric groups was 82.4% and 75.5%, respectively, with no significant disparity (*P = 0.091*). Similar findings were observed in N2 (65.0 vs. 64.3, *P = 0.162*), N3a (55.0 vs. 49.4, *P = 0.136*) and N3b (33.9 vs. 25.9, *P = 0.690*). Therefore, in each N stage, there was no significant difference in patient survival between group P and the extragastric group (Supplementary Fig. 2).

**Fig. 4:**
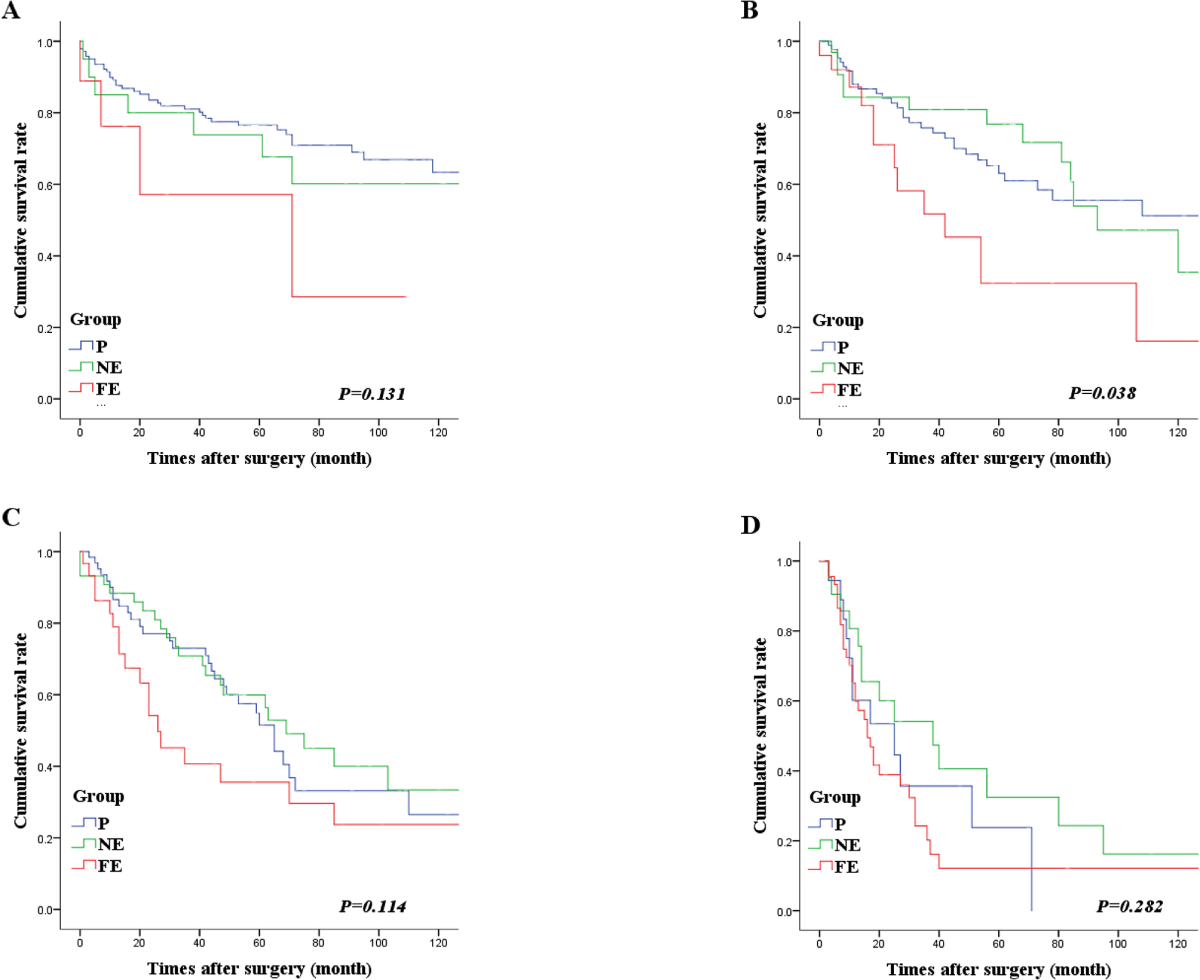
Comparison of survival according to the primary cancer location: Primary lesion at upper location. **A**: N1 stage, **B**: N2 stage, **C**: N3a stage, **D**: N3b stage. The far-extragastric group (group FE) had significantly lower overall survival, specifically in the N2 stage

**Fig. 5:**
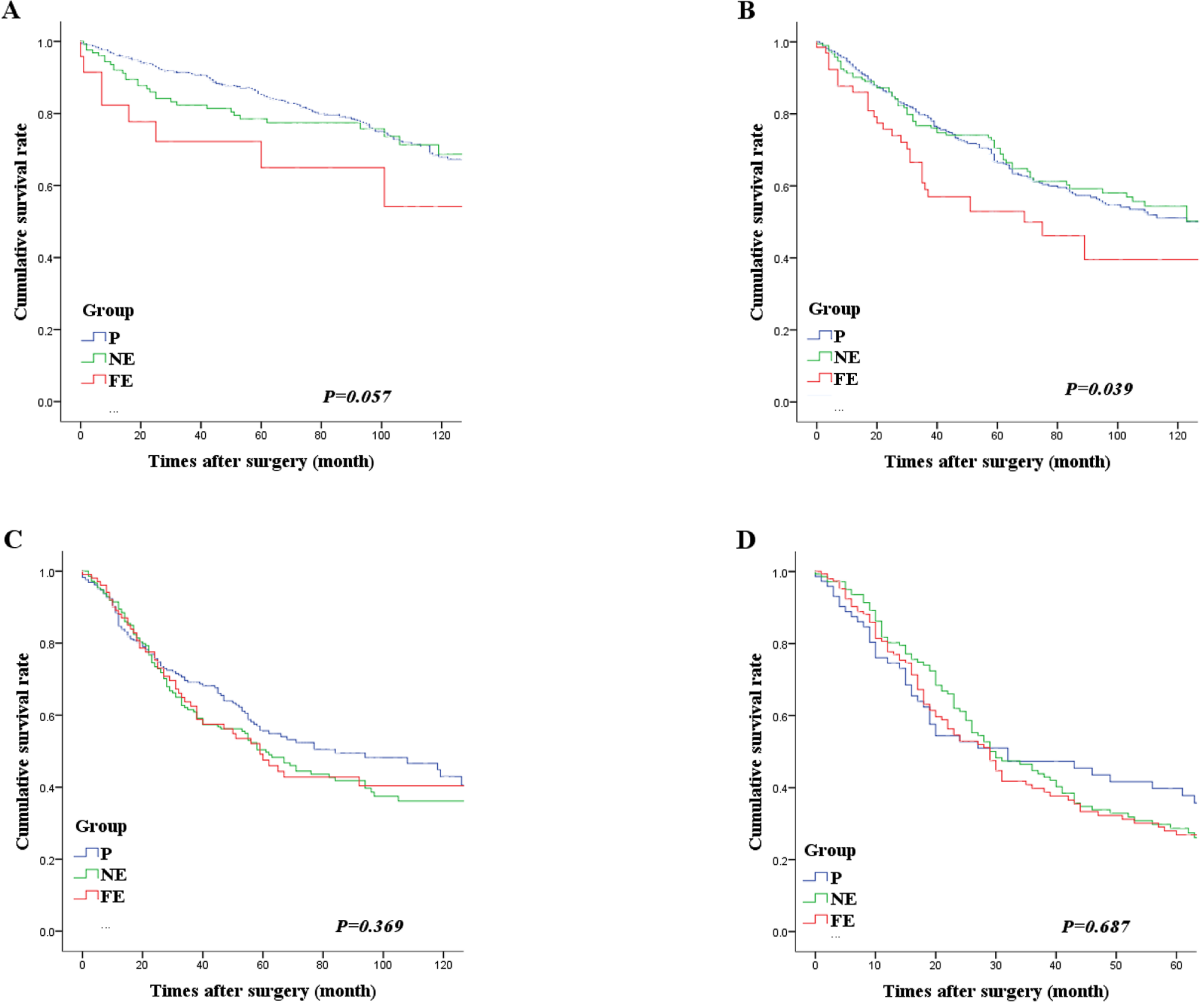
Comparison of survival according to the primary cancer location: Primary lesion at middle and/or lower location. **A**: N1 stage, **B**: N2 stage, **C**: N3a stage, **D**: N3b stage. The far-extragastric group (group FE) had significantly lower overall survival, specifically in the N2 stage

#### Comparison of overall survival between three lymph node metastasis groups

The characteristics of the Three LNM groups were compared. There were no significant differences in baseline characteristics such as age, sex, and ECOG. Group FE had a significantly higher rate of open approach and total gastrectomy, and was confirmed to have performed more lymphadenectomy of D2 or higher. Additionally, compared to group P, it was confirmed that the disease severity of groups NE and FE was relatively advanced disease (Supplementary Table 1).

The OS of the three LNM groups (P, NE, and FE) was analyzed according to the N stage. In N1, the 5YOS was 82.4% for group P, 77.9% for group NE, and 63.8% for group FE; the difference was significant (*P = 0.013*). Similarly, in N2, the 5YOS was 65.0%, 71.0%, and 46.8%, respectively (*P < 0.001*). However, the difference in OS in N3 between the three groups was not significant. These findings demonstrated that patients in group FE had significantly lower survival rates in N1 and N2 (Fig. 3).

#### Univariate and multivariate analysis of factors related to survival including three lymph node Metastasis groups

In the univariate analysis for the N1 stage, significant factors included age, sex, ECOG, BMI, approach method, lymphadenectomy extent, T stage, TNM stage, and LNM group. Through multivariate analysis, it was confirmed that the hazard ratio (HR) of group FE compared to group P was 2.457, surpassing other significant baseline variables such as age, sex, ECOG, and BMI. In the multivariate analysis of the N2 stage, group FE was established as a statistically significant factor, along with age, ECOG, and TNM stage. However, it did not exhibit significance in N3a and N3b stages (HR, CI, p-value; 1.024, 0.767–1.366, 0.873 / 1.153, 0.884–1.506, 0.293), respectively (Table 2-1, 2-2, 2-3, 2-4).

### Comparison of survival according to cancer location

We conducted an analysis according to the primary cancer location. The lesions were classified as upper, middle, or lower lesions. Subsequently, we compared the survival outcomes among the three groups. Irrespective of the cancer location, group FE had significantly lower OS rates, specifically in N2. However, for the remaining N stages except N2, there were no significant difference in survival based on the specific cancer location in relation to the MLN groups (Figs. 4 and 5).

### Comparison of survival according to extent of Surgery

We also conducted an analysis based on the extent of surgery and lymph node dissection range. Firstly, when total gastrectomy was performed, the 5YOS for group P, group NE, and group FE were as follows (N1: 75.6 vs. 66.9 vs. 61.7), (N2: 59.9 vs. 68.6 vs. 36.2), and (N3a: 67.9 vs. 61.4 vs. 23.4). These results confirmed the notably lower survival rates in group FE. For patients who underwent subtotal gastrectomy, a statistically significant outcome was observed (N3a: 40.4 vs. 29.9 vs. 28.4) (Supplementary Figs. 3 and 4). Similarly, an analysis was conducted based on the extent of lymph node dissection, revealing significantly lower OS rates in group FE exclusively in the N2 stage. This trend was observed both in the range below D1 plus (N2: 71.7 vs. 62.6 vs. 31.7) and in the range above D2 (N2: 57.7 vs. 53.8 vs. 45.7) (Supplementary Figs. 5 and 6).

## Discussion

The current TNM staging system focuses on the absolute number of MLNs and does not consider the anatomical location of LNM when predicting the prognosis of gastric cancer [18, 19]. In this study, using large-scale multi-institutional big data, we aimed to evaluate the topographical characteristics of MLNs to remedy the shortcomings of current TNM staging, especially the pathological N stage. Our findings suggest that MLN location can be used as a tool for prognosis measurement that can supplement the current TNM staging of GC. As mentioned earlier, the number of MLNs influences the prognosis of patients with GC in most circumstances. However, in cases where the number of MLNs is relatively small but located far from the tumor, the anatomical location of metastasis may also impact the prognosis.

In previous studies, many authors focused on the different classifications of LNM rather than the number or topographical nodal stages of GC [20–23]. The ratio of MLNs to harvested LNs is a good alternative, as it can be used in cases of an inadequate number of harvested LNs, thus providing valuable prognostic stratification [20]. Some researchers have suggested using the logarithmic odds ratio of positive to negative LNs as a functional, up-to-date classification of LNM [24, 25]. However, these suggested novel systems cannot overcome limitations of the current TNM system because the primary background of these systems is the same numeric-based N staging. Currently, no adequate N staging system integrates the number and anatomical regions of MLNs.

The stomach is an organ in which blood is supplied by five main vessels (the right and left gastroepiploic arteries, right and left gastric arteries, and short gastric artery). The lymphatic drainage route for GC is generally multidirectional and complicated. Therefore, compared with other malignant neoplasms, it may be difficult to consider lymphatic drainage of the stomach when incorporating the anatomical location of MLNs into an adequate N staging system [10, 26]. However, the anatomical location of MLNs could be essential to compensate for the limitations of the current N-staging system, which uses only the absolute counts of metastatic nodes [19].

The current numeric N stage has gained an overwhelming advantage in terms of utility and versatility owing to its simple and objective nature. However, there are still challenges such as the lack of clear cut-offs, insufficient anatomical information regarding the extent of LNM, and variability in the surgical extent of lymph node dissection. In our study, anatomical differences in MLNs had a limited significance in advanced nodal stages, where the number of MLNs was relatively large without restrictions. However, in N1 and N2, which are characterized by a moderate number of MLNs (1–6), the anatomical location of the MLN was a significant factor. This tendency was consistently observed across various analyses, including those stratified by tumor location, extent of surgery, and lymphadenectomy, in this study. Based on this theoretical framework, excluding the prognostic value of the far-extragastric group in nodal stages characterized by a limited number of MLNs seems unwarranted.

Although the anatomical location of MLNs cannot replace the current staging system as a tool for prognostic measurement, it is worth noting that anatomical location may influence the limited MLN stage.

This study has some limitations. First, the unavoidable biases associated with longer observation periods may have resulted in changes in treatment strategies such as changes in chemotherapy regimens and surgical guidelines. However, our data was from a multi-institutional database and compensated for this discrimination. Second, the outcomes were obtained from multiple institutions in Korea. Validation in other Eastern and Western countries may be essential to generalize these results.

Despite these limitations, our study has notable strengths that distinguish it from previous studies. First, this is one of the largest studies to date to examine the impact of the number and anatomical location of LNMs on the survival of patients with GC. Conducting prospective studies on the loopholes identified in this study has numerous challenges. Of note, the current staging system was developed based on retrospective data. Second, our study is the first to use a large-scale cohort to elucidate the significance of the anatomical location of MLNs in the survival of patients with GC with a limited number of nodal metastases.

In patients with a limited number of MLNs, the location of nodal metastases should be considered when choosing the appropriate treatment approach. For example, adjuvant chemotherapy, such as the XELOX doublet, which is applied to patients with N2 or higher disease through the CLASSIC TRIAL, can be applied to N1 patients in group FE, where the OS is expected to deteriorate. Moreover, patients with a lower T stage but N2 stage, including one or more metastatic stations 10 to 12, may require more intensive treatment and intervention to improve their survival prospects.

In conclusion, the current numerical nodal staging system is the most effective for treating gastric cancer. However, it is crucial to recognize that in the N1 and N2 stages, where there is an upper limit to the number of metastatic nodes, the specific topographic characteristics of a particular lymph node station may have significant implications.

### Electronic supplementary material

Below is the link to the electronic supplementary material.

Supplementary Material 1

Supplementary Material 2

Supplementary Material 3

Supplementary Material 4

Supplementary Material 5

Supplementary Material 6

Supplementary Material 7

## Data Availability

The datasets used and/or analysed during the current study available from the corresponding author on reasonable request.

## References

[1] Sung H, Ferlay J, Siegel RL, Laversanne M, Soerjomataram I, Jemal A (2021). Global Cancer statistics 2020: GLOBOCAN estimates of incidence and Mortality Worldwide for 36 cancers in 185 countries. CA Cancer J Clin.

[2] Park SH, Kang MJ, Yun EH, Jung KW (2022). Epidemiology of gastric Cancer in Korea: Trends in Incidence and Survival based on Korea Central Cancer Registry Data (1999–2019). J Gastric Cancer.

[3] Sexton RE, Al Hallak MN, Diab M, Azmi AS (2020). Gastric cancer: a comprehensive review of current and future treatment strategies. Cancer Metastasis Rev.

[4] Wittekind C (2015). The development of the TNM classification of gastric cancer. Pathol Int.

[5] Ichikura T, Tomimatsu S, Uefuji K, Kimura M, Uchida T, Morita D (1999). Evaluation of the New American Joint Committee on Cancer/International Union against cancer classification of lymph node Metastasis from gastric carcinoma in comparison with the Japanese classification. Cancer.

[6] Fujii K, Isozaki H, Okajima K, Nomura E, Niki M, Sako S (1999). Clinical evaluation of lymph node Metastasis in gastric cancer defined by the fifth edition of the TNM classification in comparison with the Japanese system. Br J Surg.

[7] Nio Y, Tsubono M, Kawabata K, Masai Y, Hayashi H, Meyer C (1993). Comparison of survival curves of gastric cancer patients after Surgery according to the UICC stage classification and the General rules for gastric Cancer study by the Japanese Research Society for gastric cancer. Ann Surg.

[8] Kikuchi S, Sakakibara Y, Sakuramoto S, Kobayashi N, Shimao H, Mieno H (2001). Recent results in the surgical treatment of gastric cancer according to the Japanese and TNM classification. Anticancer Res.

[9] Hayashi H, Ochiai T, Suzuki T, Shimada H, Hori S, Takeda A (2000). Superiority of a new UICC-TNM staging system for gastric carcinoma. Surgery.

[10] Maruyama K, Sasako M, Kinoshita T, Sano T, Katai H (1999). Can sentinel node biopsy indicate rational extent of lymphadenectomy in gastric cancer Surgery? Fundamental and new information on lymph-node dissection. Langenbecks Arch Surg.

[11] Kim TH, Kim IH, Kang SJ, Choi M, Kim BH, Eom BW (2023). Korean practice guidelines for gastric Cancer 2022: an Evidence-based, Multidisciplinary Approach. J Gastric Cancer.

[12] Japanese Gastric Cancer Treatment Guidelines 2021 (6th edition). Gastric Cancer. 2023;26(1):1–25. 10.1007/s10120-022-01331-8 PMC9813208 36342574

[13] Moertel CG, Lavin PT (1979). Phase II-III chemotherapy studies in advanced gastric cancer. Eastern Cooperative Oncology Group. Cancer Treat Rep.

[14] Dindo D, Demartines N, Clavien PA (2004). Classification of Surgical Complications: a new proposal with evaluation in a cohort of 6336 patients and results of a survey. Ann Surg.

[15] Amin MB, Greene FL, Edge SB, Compton CC, Gershenwald JE, Brookland RK (2017). The Eighth Edition AJCC Cancer staging Manual: continuing to build a bridge from a population-based to a more personalized approach to cancer staging. CA Cancer J Clin.

[16] Bulent Cavit Y, Okan Murat A, Ilyas Hakan O, Nabil I (2011). Lymph node dissection in gastric carcinoma. Management of gastric Cancer.

[17] Degiuli M, De Manzoni G, Di Leo A, D’Ugo D, Galasso E, Marrelli D (2016). Gastric cancer: current status of lymph node dissection. World J Gastroenterol.

[18] Jeong O, Jung MR, Kang JH (2021). Prognostic value of the Anatomic Region of Metastatic Lymph Nodes in the current TNM staging of gastric Cancer. J Gastric Cancer.

[19] Choi YY, An JY, Katai H, Seto Y, Fukagawa T, Okumura Y (2016). A lymph node staging system for gastric Cancer: a hybrid type based on Topographic and Numeric systems. PLoS ONE.

[20] Marchet A, Mocellin S, Ambrosi A, Morgagni P, Garcea D, Marrelli D (2007). The ratio between metastatic and examined lymph nodes (N ratio) is an Independent prognostic factor in gastric cancer regardless of the type of lymphadenectomy: results from an Italian multicentric study in 1853 patients. Ann Surg.

[21] Kong SH, Lee HJ, Ahn HS, Kim JW, Kim WH, Lee KU (2012). Stage migration effect on survival in gastric cancer Surgery with extended lymphadenectomy: the reappraisal of positive lymph node ratio as a proper N-staging. Ann Surg.

[22] Cheong JH, Hyung WJ, Shen JG, Song C, Kim J, Choi SH (2006). The N ratio predicts recurrence and poor prognosis in patients with node-positive early gastric cancer. Ann Surg Oncol.

[23] Xu DZ, Geng QR, Long ZJ, Zhan YQ, Li W, Zhou ZW (2009). Positive lymph node ratio is an Independent prognostic factor in gastric cancer after d2 resection regardless of the examined number of lymph nodes. Ann Surg Oncol.

[24] Gu P, Deng J, Sun Z, Wang Z, Wang W, Liang H (2021). Superiority of log odds of positive lymph nodes (LODDS) for prognostic prediction after gastric cancer Surgery: a multi-institutional analysis of 7620 patients in China. Surg Today.

[25] Sun Z, Xu Y, Li de M, Wang ZN, Zhu GL, Huang BJ (2010). Log odds of positive lymph nodes: a novel prognostic indicator superior to the number-based and the ratio-based N category for gastric cancer patients with R0 resection. Cancer.

[26] Biondi A, Hyung WJ (2011). Seventh edition of TNM classification for gastric cancer. J Clin Oncol.

