# Supplementary figures and images for: Refining gastric cancer staging: examining the interplay between number and anatomical location of metastatic lymph nodes - a retrospective multi-institutional study
Source: BMC Cancer. 2023 Dec 5;23:1192. doi: 10.1186/s12885-023-11653-0 (PMC10699030; doi:10.1186/s12885-023-11653-0)

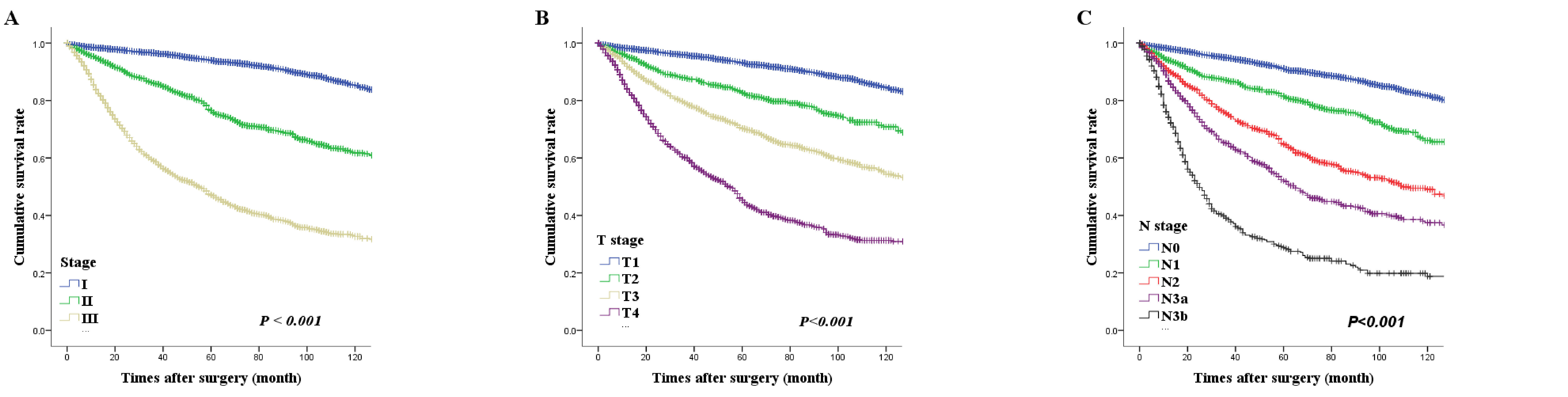

Supplement: Supplementary file 1 — Supplementary Material 1 [file 12885_2023_11653_MOESM1_ESM.tif]

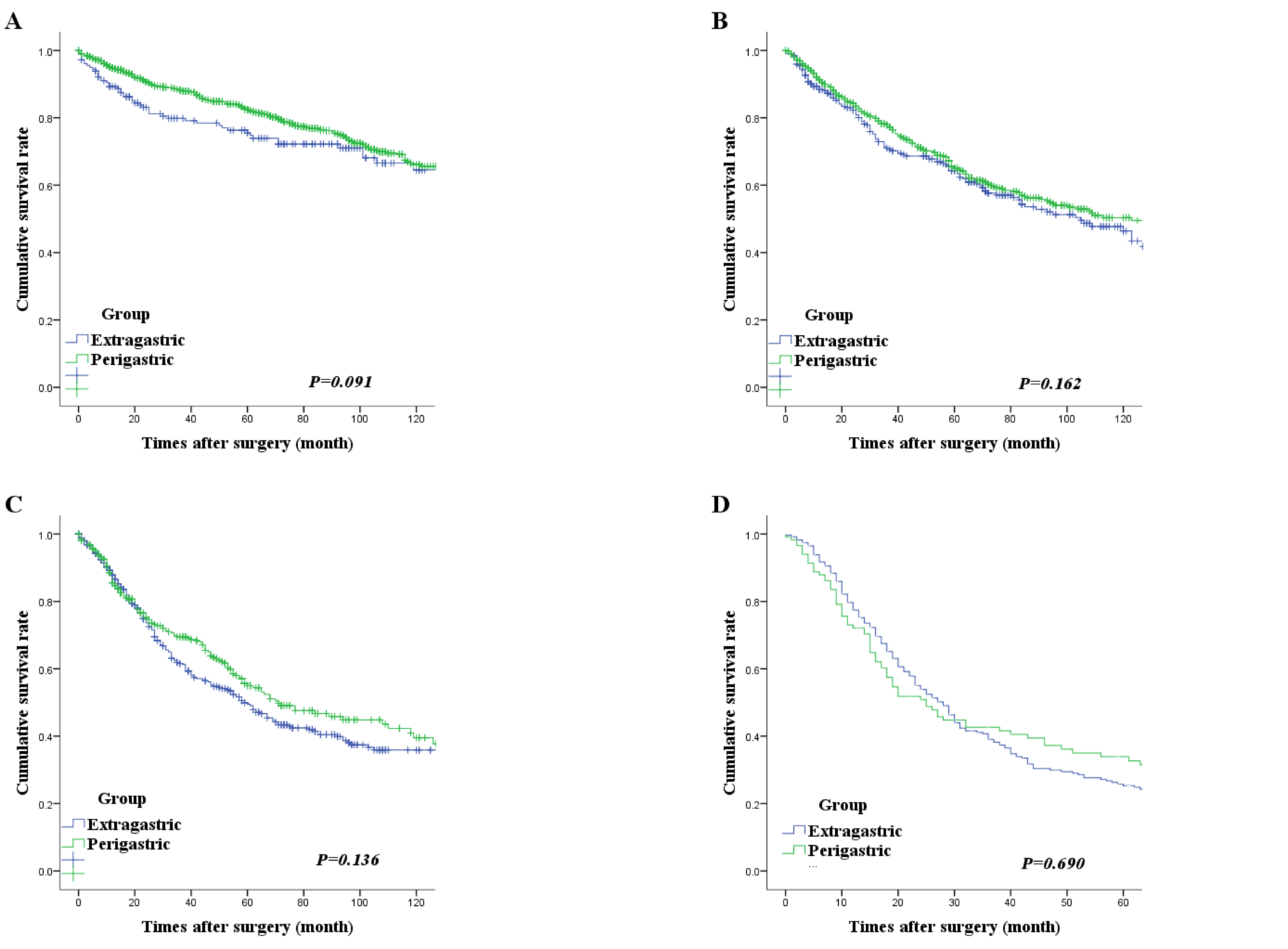

Supplement: Supplementary file 2 — Supplementary Material 2 [file 12885_2023_11653_MOESM2_ESM.tif]

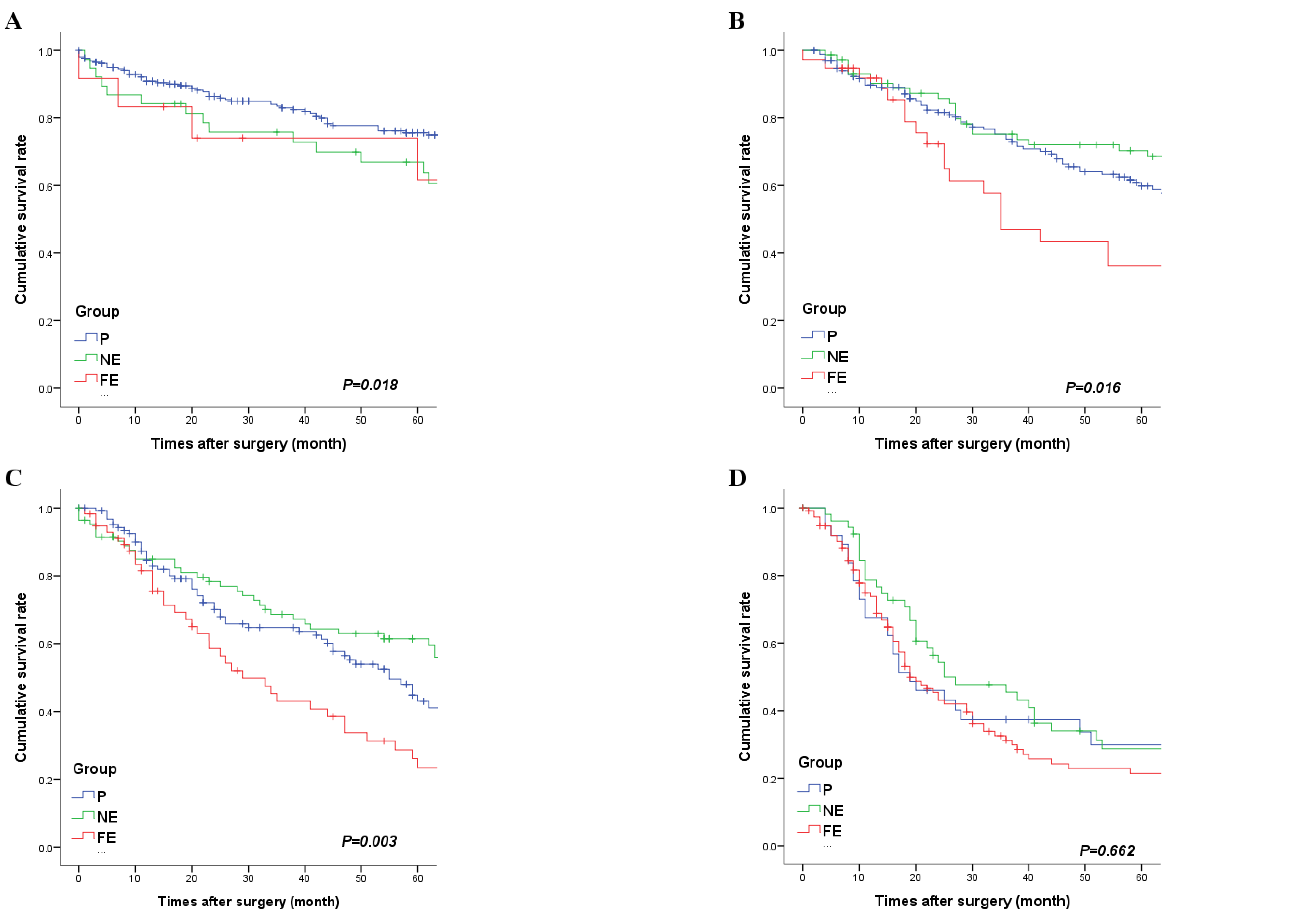

Supplement: Supplementary file 3 — Supplementary Material 3 [file 12885_2023_11653_MOESM3_ESM.tif]

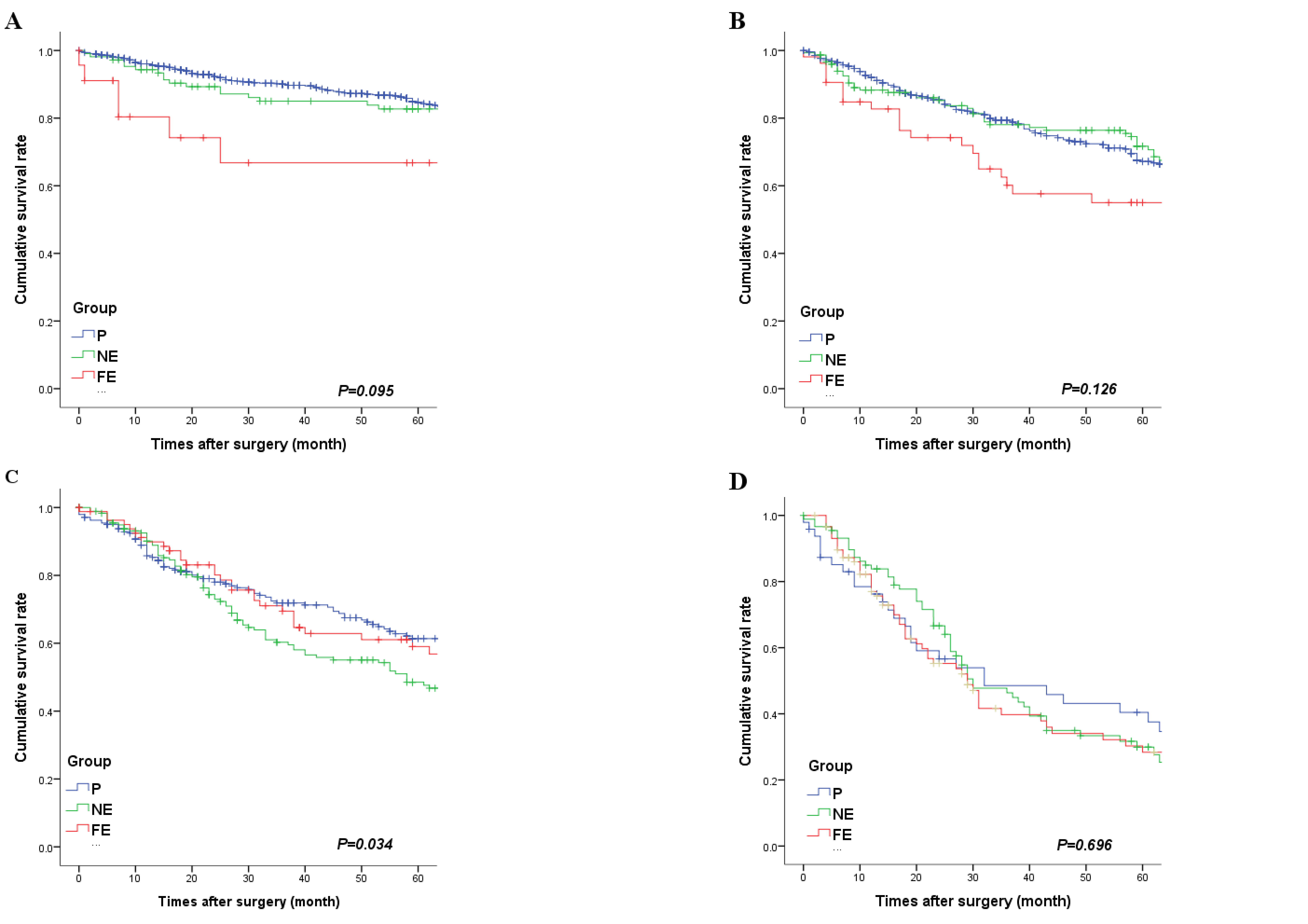

Supplement: Supplementary file 4 — Supplementary Material 4 [file 12885_2023_11653_MOESM4_ESM.tif]

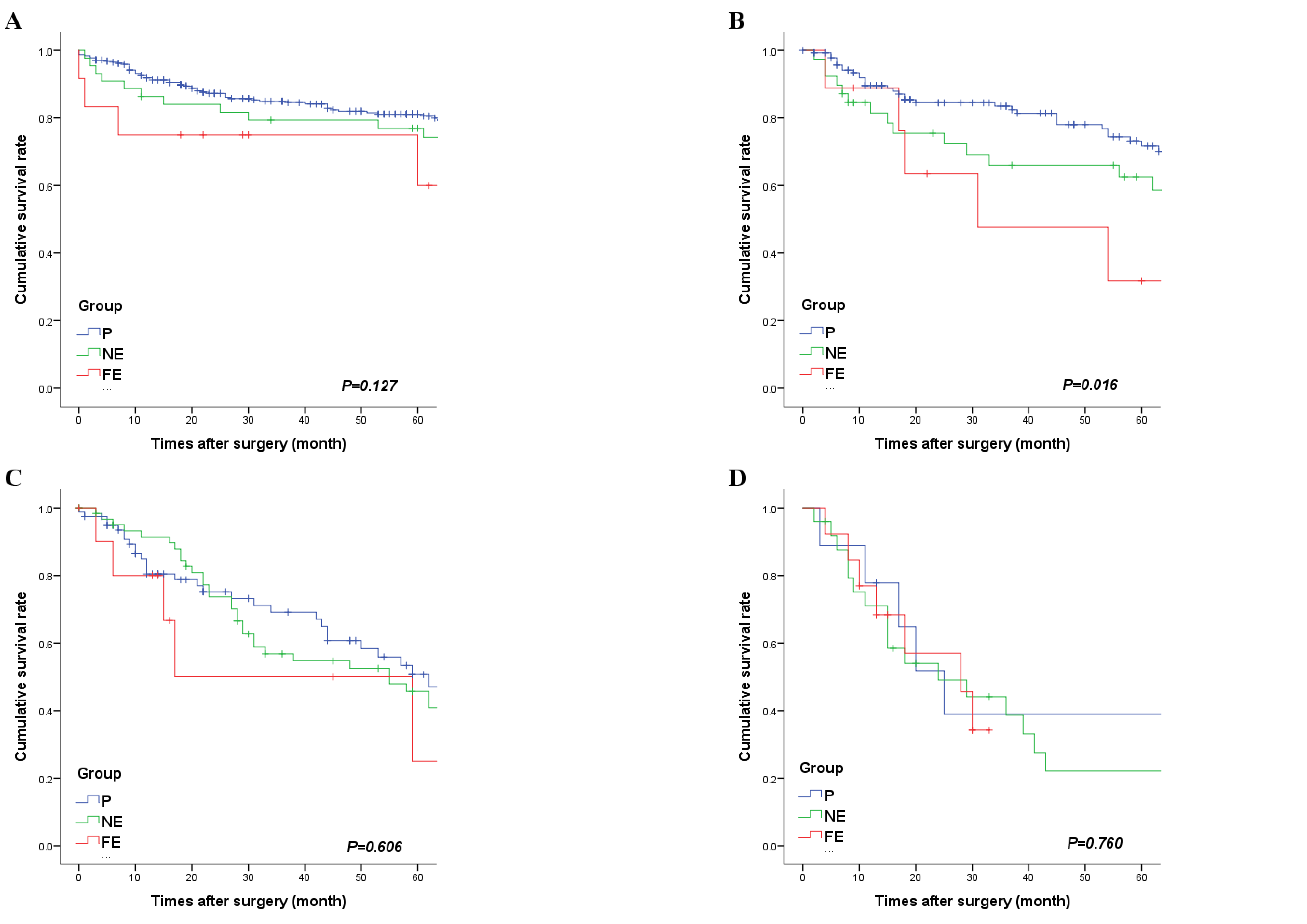

Supplement: Supplementary file 5 — Supplementary Material 5 [file 12885_2023_11653_MOESM5_ESM.tif]

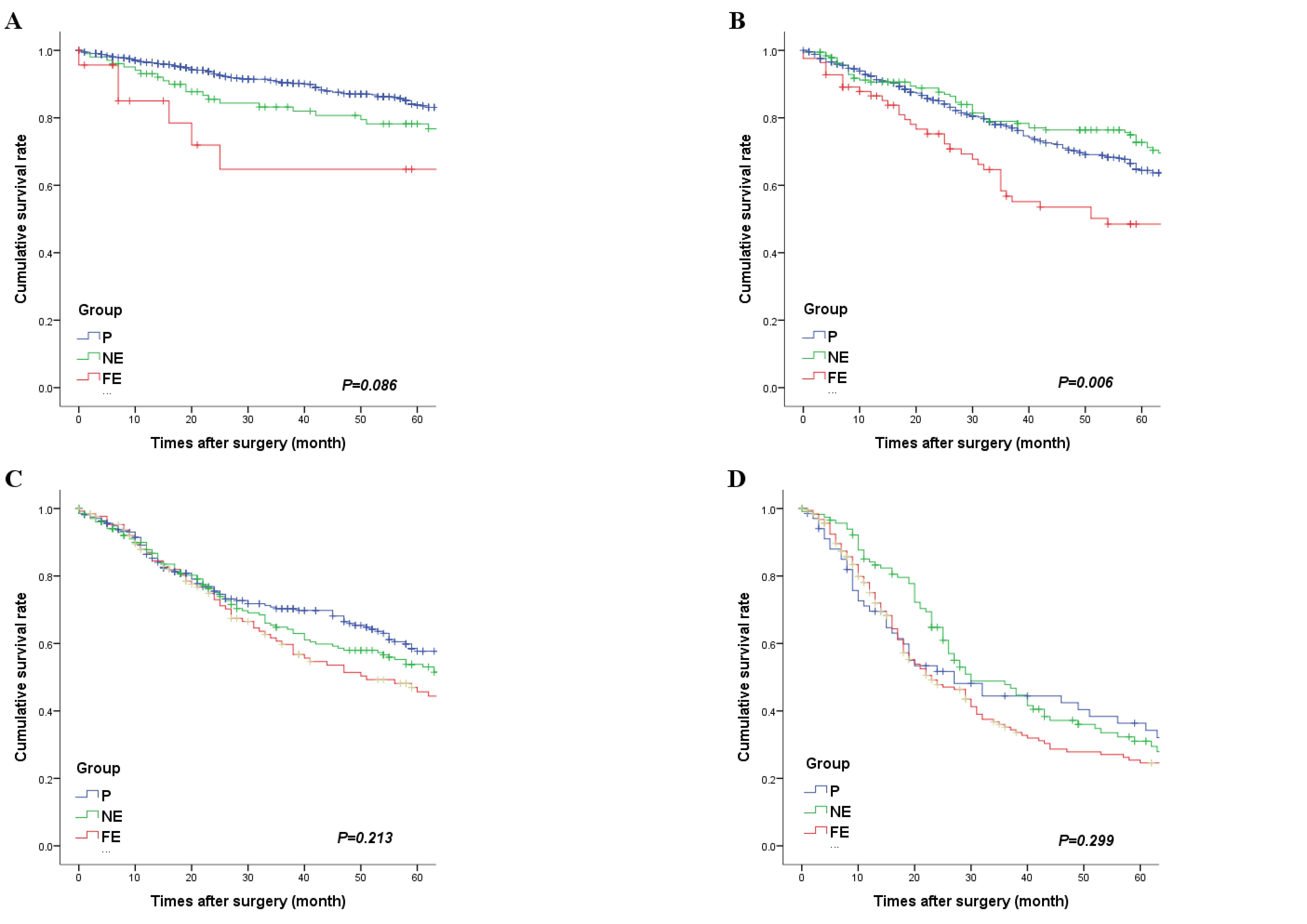

Supplement: Supplementary file 6 — Supplementary Material 6 [file 12885_2023_11653_MOESM6_ESM.tif]
