# Supplementary material for: Refining gastric cancer staging: examining the interplay between number and anatomical location of metastatic lymph nodes - a retrospective multi-institutional study
Source: BMC Cancer. 2023 Dec 5;23:1192. doi: 10.1186/s12885-023-11653-0 (PMC10699030; doi:10.1186/s12885-023-11653-0)
Supplement: Supplementary file 7 — Supplementary Material 7 [file 12885_2023_11653_MOESM7_ESM.docx]

**Figure** **legends**

**Supplementary Figure 1** Overall survival was compared according to disease severity. A: Pathologic stage, B: Tumor stage, C: Nodal stage. A decline in survival was observed with increasing disease severity.

**Supplementary Figure 2** Survival comparison between Perigastric and Extragastric groups. A: N1 stage, B: N2 stage, C: N3a stage, D: N3b stage. There was no significant difference in patient survival between the perigastric and extragastric groups in each N stage.

**Supplementary Figure 3** Survival comparison between three metastatic lymph node groups of total gastrectomy. A: N1 stage, B: N2 stage, C: N3a stage, D: N3b stage.

**Supplementary Figure 4** Survival comparison between three metastatic lymph node groups of subtotal gastrectomy. A: N1 stage, B: N2 stage, C: N3a stage, D: N3b stage.

**Supplementary Figure 5** Survival comparison between three metastatic lymph node groups of D1 plus lymphadenectomy or less. A: N1 stage, B: N2 stage, C: N3a stage, D: N3b stage.

**Supplementary Figure 5** Survival comparison between three metastatic lymph node groups of D2 lymphadenectomy or more. A: N1 stage, B: N2 stage, C: N3a stage, D: N3b stage.

**Supplementary Table 1** Patient characteristics across the three LNM groups

| **Variables, n(%)** | **Group P**  **(N = 2,267)** | **Group NE**  **(N = 813)** | **Group FE**  **(N = 511)** |  | **P-value** |  |
| --- | --- | --- | --- | --- | --- | --- |
| **Age (years)** |  |  |  |  | 0.267 |  |
| < 65 | 1213 (53.5%) | 434 (53.4%) | 293 (57.3%) |  |  |  |
| ≥ 65 | 1054 (46.5%) | 379 (46.6%) | 218 (42.8%) |  |  |  |
| **Sex** |  |  |  |  | 0.751 |  |
| Male | 1532 (67.6%) | 541 (66.5%) | 350 (68.5%) |  |  |  |
| Female | 735 (32.4%) | 272 (33.5%) | 161 (31.5%) |  |  |  |
| **ECOG** |  |  |  |  | 0.350 |  |
| 0-1 | 2144 (94.6%) | 768 (94.4%) | 484 (94.7%) |  |  |  |
| ≥ 2 | 166 (4.7%) | 38 (4.7%) | 22 (4.3%) |  |  |  |
| N/A | 17 (0.7%) | 7 (0.9%) | 5 (1.0%) |  |  |  |
| **Preoperative BMI  (kg/m^2^)** |  |  |  |  | 0.005 |  |
| < 23 | 812 (45.8%) | 263 (47.7%) | 228 (54.7%) |  |  |  |
| ≥ 23 | 962 (54.2%) | 288 (52.3%) | 189 (45.3%) |  |  |  |
| **Approach** |  |  |  |  | < 0.001 |  |
| MIS | 933 (41.2%) | 197 (24.2%) | 92 (18.0%) |  |  |  |
| Open | 1269 (56.0%) | 581 (71.5%) | 404 (79.1%) |  |  |  |
| N/A | 65 (2.8%) | 35 (4.3%) | 15 (2.9%) |  |  |  |
| **Resection** |  |  |  |  | < 0.001 |  |
| STG | 1623 (71.6%) | 542 (66.7%) | 262 (51.3%) |  |  |  |
| TG | 627 (27.7%) | 263 (32.3%) | 244 (47.7%) |  |  |  |
| Others | 17 (0.7%) | 8 (1.0%) | 5 (1.0%) |  |  |  |
| **Lymphadenectomy** |  |  |  |  | < 0.001 |  |
| D1+ ↓ | 569 (25.1%) | 185 (22.8%) | 52 (10.2%) |  |  |  |
| D2 ↑ | 1640 (72.3%) | 628 (77.2%) | 459 (89.8%) |  |  |  |
| N/A | 58 (2.6%) | 0 (0.0%) | 0 (0.0%) |  |  |  |
| **Reconstruction** |  |  |  |  | < 0.001 |  |
| B-Ⅰ | 269 (11.9%) | 81 (10.0%) | 31 (6.1%) |  |  |  |
| B-Ⅱ | 1248 (55.1%) | 432 (53.1%) | 219 (42.9%) |  |  |  |
| RY | 630 (27.8%) | 239 (29.4%) | 224 (43.8%) |  |  |  |
| Others | 120 (5.2%) | 61 (7.5%) | 37 (7.2%) |  |  |  |
| **pT stage** |  |  |  |  | < 0.001 |  |
| T1 | 575 (25.4%) | 120 (14.8%) | 40 (7.8%) |  |  |  |
| T2 | 339 (15.0%) | 106 (13.0%) | 42 (8.2%) |  |  |  |
| T3 | 647 (28.5%) | 246 (30.3%) | 129 (25.2%) |  |  |  |
| T4 | 706 (31.1%) | 341 (41.9%) | 300 (58.7%) |  |  |  |
| **pN stage** |  |  |  |  | < 0.001 |  |
| N1 | 1096 (48.4%) | 146 (18.0%) | 35 (6.8%) |  |  |  |
| N2 | 654 (28.8%) | 226 (27.8%) | 92 (18.0%) |  |  |  |
| N3a | 371 (16.4%) | 267 (32.8%) | 140 (27.5%) |  |  |  |
| N3b | 146 (6.4%) | 174 (21.4%) | 244 (47.7%) |  |  |  |
| **pTMN stage** |  |  |  |  | < 0.001 |  |
| I | 610 (26.9%) | 60 (7.4%) | 14 (2.7%) |  |  |  |
| Ⅱ | 694 (30.6%) | 161 (19.8%) | 32 (6.3%) |  |  |  |
| Ⅲ | 963 (42.5%) | 592 (72.8%) | 465 (91.0%) |  |  |  |

*Abbreviations*: *LNM* lymph node metastasis, *ECOG* Eastern Cooperative Oncology Group performance status, *N/A* not applicable, *BMI* body mass index, *MIS* minimal invasive surgery, *STG* subtotal gastrectomy, *TG* total gastrectomy
